# Situational simulation teaching effectively improves dental students’ non-operational clinical competency and objective structured clinical examination performance

**DOI:** 10.1186/s12909-024-05546-4

**Published:** 2024-05-14

**Authors:** Ju-Hui Wu, Pei Chen Lin, Kun-Tsung Lee, Hsin-Liang Liu, Peih-Ying Lu, Chen-Yi Lee

**Affiliations:** 1 Department of Oral Hygiene, College of Dental Medicine, Kaohsiung Medical University, Kaohsiung, Taiwan No. 100, Shih-Chuan 1st Road, 80708; 2 Department of Dentistry, Kaohsiung Medical University Hospital, Kaohsiung, Taiwan; 3 Center for Medical Education and Humanizing Health Professional Education, Kaohsiung Medical University Hospital, Kaohsiung, Taiwan; 4 Graduate Institute of Adult Education, National Kaohsiung Normal University, Kaohsiung, Taiwan; 5 Department of Emergency Medicine, Kaohsiung Medical University Hospital, Kaohsiung Medical University, Kaohsiung, Taiwan; 6 School of Medicine, Kaohsiung Medical University, Kaohsiung, Taiwan; 7 College of Humanities and Social Sciences, Kaohsiung Medical University, Kaohsiung, Taiwan; 8 Department of Medical Research, Kaohsiung Medical University Hospital, Kaohsiung, Taiwan

**Keywords:** Clinical simulation education, Simulated patient, Objective structured clinical examination, Undergraduate dental students

## Abstract

**Background:**

Appropriate communication with dental patients enhances treatment outcomes and patient satisfaction. Implementing simulated patient interviews courses can improve patient-centered care and reduce conflict during clerkship training. Therefore, this study explored the relationship among student participation in a situational simulation course (SSC), academic performance, clerkship performance, and objective structured clinical examination (OSCE) performance.

**Methods:**

This study was conducted with a sample of fifth-year dental students undergoing clerkship training. After implementing a situational simulation course to investigate the relationship among participation in SSC, academic performance, clerkship performance, and OSCE performance, a path analysis model was developed and tested.

**Results:**

Eighty-seven fifth-year dental students were eligible for the SSC, and most (*n* = 70, 80.46%) volunteered to participate. The path analysis model revealed that academic performance had a direct effect on OSCE performance (β = 0.281, *P* = 0.003) and clerkship performance (β = 0.441, *P* < 0.001). In addition, SSC teaching had a direct effect on OSCE performance (β = 0.356, *P* < 0.001).

**Conclusions:**

SSCs can enhance dental students’ non-operational clinical competency and OSCE performance effectively. Simulated patient encounters with feedback, incorporated into the dental curricula, have led to improved communication. Based on our findings, we suggest implementing SSC teaching before the OSCE to improve communication and cognitive skills.

## Background

Effective communication is vital to ensure quality clinical practice. Appropriate communication with dental patients may enhance diagnostic efficiency [1], support clinical decision-making [2], decrease dental patient anxiety [3], increase positive clinical outcomes, and improve oral hygiene [4], periodontal compliance [5], and patient-clinician satisfaction [6]. In a scoping review, most studies presented evidence that supported the use of simulated patients in healthcare training, suggesting it may support the development of clinical competences among students [7]. Clinical competences encompass technical skills, non-technical skills, and cognitive skills. The core competencies of Taiwanese dental graduates are categorized into operational and non-operational categories [8], with the latter set encompassing non-technical skills (such as communication) as well as cognitive skills (including decision-making and clinical reasoning), which are essential parts of dental training and practice [8]. However, 26.5% of the fifth-year dental students performed poorly at interpersonal communication during the OSCE assessment prior to their internship course [9], suggesting a need to teach patient-centered communication. Enhancing communication skills development within clerkship training facilitated by dental educators is imperative for improving students’ non-technical skills.

A review of teaching and assessment methods in dental practice revealed that educators use a blend of passive and interactive strategies to support communication training [10]. Interactive pedagogical techniques include role-playing, simulated patient encounters, interactions with standardized patients (SPs), actual patient interviews, small-group activities, and video-based exercises. A systematic review revealed that the fundamental principles for effective communication training encompass active learning strategies, such as incorporating practical exercises, clinically relevant scenarios, self-assessment tools for students, use of videotapes, and involvement of patient actors [11, 12]. However, dental school curricula often have insufficient skill-activating programs [12].

Communication skills can be taught in both simulated and actual clinical environments. However, it is essential to incorporate patient-centered care into dental clerkship education, with simulations emerging as a recommended strategy for imparting safe clinical practice. Educational simulations are typically categorized into four groups: physical, iterative, procedural, and situational [13]. Situational simulations typically simulate human behavior, emphasizing the attitudes exhibited by individuals or groups within specific contexts. These simulations frequently utilize role-playing to enable students to investigate various alternatives and decision-making processes [13]. In medical training, situational simulation teaching is an educational approach with structured scenarios and SPs that simulate the essential aspects of a clinical situation so that a student can practice skills and thereby consolidate learning. This approach has been shown to improve students’ clinical competence, helping develop in a safe and supportive environment [14–16].

There are several learning theories, such as constructivism, cognitivism, experiential learning, active learning, and Miller’s Pyramid, underpinning the design and implementation of simulation-based education [17]. Constructivism defines knowledge as built upon an existing foundation through interaction between learners and their environment. Experiential learning posit that learning occurs through experience and reflection on action within simulations. Briefing and debriefing activate learners’ prior knowledge by enquiring about their current level of knowledge or previous exposure to similar situations. Debriefing, an essential element in simulations [18], serves as a tool for improving student performance through reflection [19].

The process of simulation-based education, which is effective in focusing on outcome-based objectives, includes pre-briefing, simulation, and debriefing [16]. It has been used to train communication skills and has powerful effects on learner-centered outcomes in health profession education [20, 21]. In dental education, simulated patient encounters with feedback may improve trainee communication skills [22, 23]. However, SPs tend to be underutilized often due to cost and resource constraints. Furthermore, McKenzie et al. identified the diminishing effectiveness of SP simulation education over time [24]. Nevertheless, many dental school graduates face challenges in progressing to clinical clerkship, which aims to prepare them for the rigors and realities of independent practice. Dental educators contend that effective communication is a fundamental clinical skill that must be part of the core curriculum. However, communication skills training is often relegated to lectures or passive learning methods, offering limited opportunities for hands-on and skill-based practices [12, 24]. Finally, previous studies have recommended introducing doctor-patient communication courses in Taiwan to teach communication skills [25].

Therefore, the primary objective of our curriculum was to teach dental students to effectively convey medical information to patients while exemplifying the qualities of sensitivity, honesty, and empathy. Situational simulation teaching was adopted in family dentistry courses, and fifth-year dentistry students were invited to participate in the course voluntarily. The present study had two aims: (1) to evaluate students’ performance and attitude toward situational simulation-based learning in improving communication and cognitive skills, and (2) to explore the relationship between students’ participation in situational simulation course (SSC), academic performance, clerkship performance, and OSCE performance.

## Methods

**Fig. 1:**
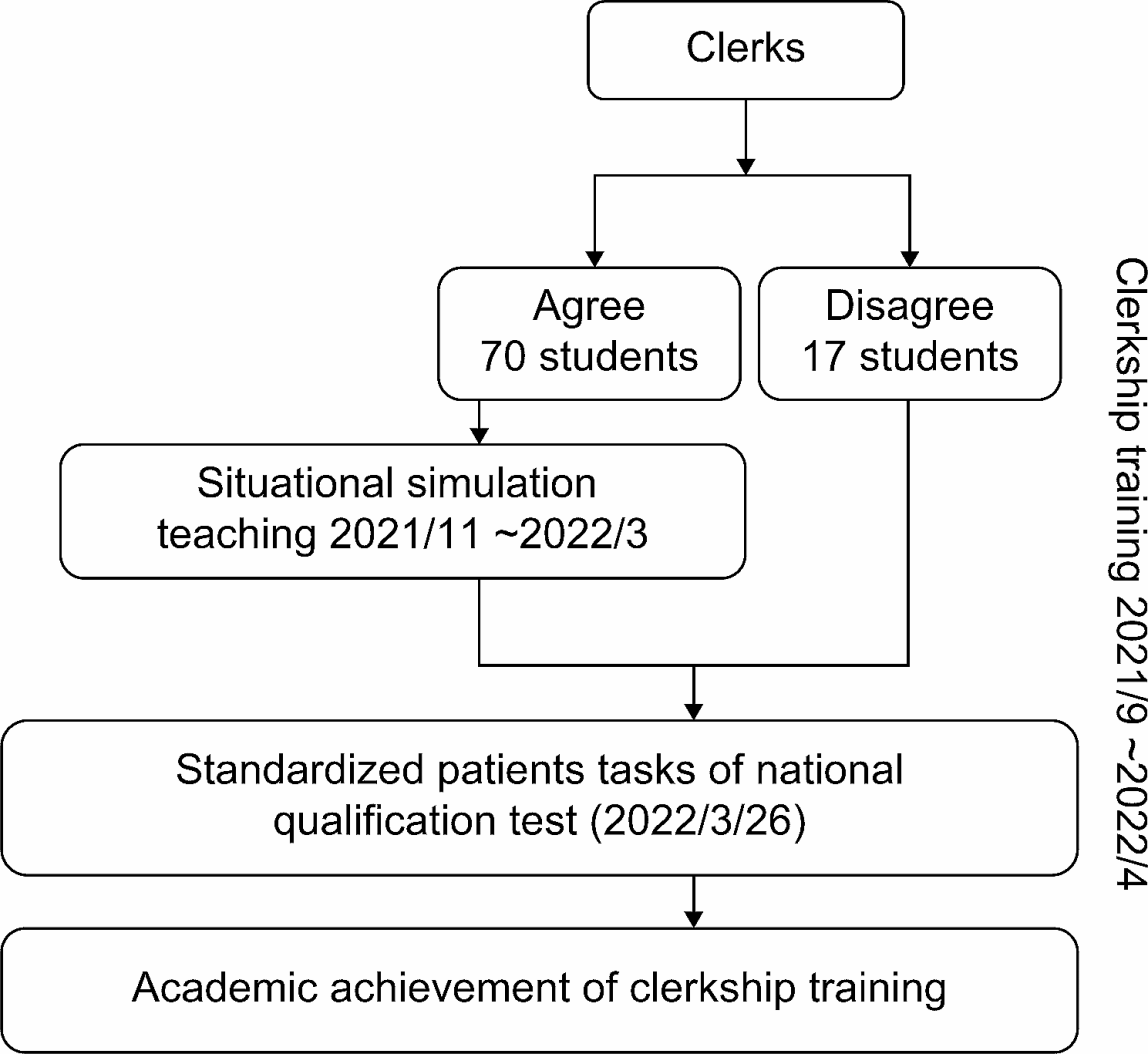
Flowchart of the research process

This study was conducted on a sample of fifth-year dental students during clerkship training. Eighty-seven fifth-year dental students eligible for the SSC were recruited for this study. Most students (*n* = 70, 80.46%) volunteered to participate in the simulations. Before completing their training, all students must participate in a national qualification test administered by the Association for Dental Sciences of the Republic of China. The national qualification test includes an OSCE, a written examination, and a skill operation examination. The test results of the SPs tasks were adopted as outcome variables in this study to evaluate the long-term effects of the SSC. The SPs task station was scored with 12 items, each rated on a scale of 0 to 2, where 0 = did not meet the requirements, 1 = partially met the requirements, and 2 = met the requirements. The sum of the raw scores were then scaled using the formula: raw score/total score × 100. A student was required to obtain 50 points (out of 100 points) to pass the station. Additionally, following completion of clerkship training, tutors of each specialty assessed the participants’ clerkship performances, and an average assessment was adopted as the clerkship performance score. Furthermore, the average score from the first semester of the second year to the first semester of the fifth year of the students was considered as their academic performance score, excluding first-grade scores, as these courses primarily cover introductory science (e.g., general biology, general chemistry, etc.) and liberal arts education. Finally, the clerkship and academic performances were included in this study to explore the potential cause-effect relationship of situational simulation learning. A flowchart of the research process is shown in Fig. 1.

### Ethics

This study was approved by the Institutional Review Board of the Kaohsiung Medical University Hospital (Approval Letters: KMUHIRB-SV(II)-20,210,057 and KMUHIRB-SV(I)-20,220,047). All the participants provided written informed consent. The clerkship performance scores of students were evaluated by a supervisor at each station, and the supervisors did not know whether the students have participated in the SSC to help reduce bias. The procedures, possible discomforts or risks, and potential benefits were explained fully to the participants involved, and their informed consent was obtained prior to the study. The study was performed in accordance with the Declaration of Helsinki.

### Description of the situational simulation teaching courses

The SSC intervention was designed to train dental students in non-operational clinical competency and considered students’ backgrounds, experiences, and learning environments. The skills selected for this intervention included history taking and information giving, oral appliance instruction, image interpretation, and communication among professionals with SBAR (situation, background, assessment, and recommendation) [26]. Each group of two students participated in four 25-min scenarios (Table 1), which were conducted at the dental clinic (in situ).

**Table 1:** Design of situational simulation teaching courses

Stations	History taking and differential diagnosis with disabilities	Managing sudden cardiac arrest in the dental office	Oral hygiene instruction for patients with oral appliances	Oral care before denosumab injection
Learning objectives	• Safe wheelchair transfer • History taking and differential diagnosis • Appropriate referrals to oral and maxillofacial surgeons	• Call the emergency response team • Cardiopulmonary resuscitation/Basic life support	• Explanation and oral hygiene instruction with oral appliances	• Explanation and oral image condition • Oral hygiene instruction

The SPs were provided with a comprehensive script outlining the simulated clinical scenarios, including potential responses and questions to be anticipated. The phases of the SSC intervention included preparation, briefing (pre-briefing), simulation activity, feedback, debriefing, evaluation, and reflection. Learners’ engagement in the simulation-based learning process entails a preparatory phase wherein foundational knowledge acquisition is necessary to participate in the simulation experience actively. Subsequently, this preparation was supplemented by a briefing (pre-briefing) session that imparted crucial information encompassing the intended learning outcomes of the session. Following the briefing (pre-briefing), learners proceeded to the simulation-based learning activity, culminating in the feedback, debriefing, and reflective phases.

### Participant characteristics

Data on the students’ characteristics, including sex, age, academic performance (average scores from the first semester of second to the first semester of fifth years), OSCE performance (total score) and outcome (pass/fail) from the qualification test, and clerkship performance, were collected from their learning histories.

### Outcome assessment of SSC

Entrustment rating scales for each station were developed by the author (HLL) based on Holzhausen et al. [27]. The entrustment level was rated on five levels: 1 = in co-activity with the supervisor, 2 = the supervisor is present and steps in if needed, 3 = performed with supervision available within minutes and key findings double-checked, 4 = performed with supervision available but from a distance and key findings double-checked, and 5 = performed with remote monitoring and key findings reviewed. The supervisors evaluated the student’s performance in the SSC; if any station was considered level 2, the course outcome was rated as a failure.

### Attitude toward SSC

The author (HLL) designed the questionnaire based on literature reviews of simulation-based learning [28, 29]. Subsequently, a team of multidisciplinary professionals, consisting of six experts, evaluated the questionnaire for its face validity and implemented any necessary adjustments. The scale consisted of five dimensions: five items related to situational simulation planning, six related to supervision and teaching, four related to improving self-efficacy, five related to interdisciplinary learning, and three related to professionalism. The items were answered using a 5-point Likert scale ranging from 1 (strongly disagree) to 5 (strongly agree) points, and the sum scores of each subscale were obtained. The internal consistency of the subscales in this sample was 0.853, 0.903, 0.844, 0.849, and 0.886. Furthermore, students were asked to answer a question regarding their ability to improve after the course.

### Statistical analysis

Data were analyzed using the IBM Statistical Package for Social Sciences for Windows (version 20.0; SPSS Inc., Armonk, New York, USA). Statistical significance was set at P-values of < 0.05. The sample characteristics are presented using descriptive statistics. For univariate analysis, age was categorized into two groups (23–24 years and > 25 years) because students above 25 years of age showed a strong desire to be a dentist, as they had taken the College Entrance Examination more than two times or even retaken it after earning a bachelor’s degree from another institution. Academic performance was grouped based on the mean score for univariate analyses when treating them as categorical variables. Differences in sample characteristics, participation in SSC, academic performance, OSCE performance, and clerkship performance were assessed using an independent sample t-test or chi-square test.

To investigate the relationships between participation in SSC (yes/no), academic performance (continuous variable), clerkship performance (continuous variable), and OSCE performance (continuous variable), a path analysis model was developed and tested using AMOS 26. Rigorous evaluation criteria were adopted to ensure an adequate model fit. A χ^2^ test was chosen as the statistical test of model fit (α = 0.05). The goodness-of-fit index (GFI), comparative fit index (CFI), Tucker-Lewis index (TLI), and root mean square error of approximation (RMSEA) were used to evaluate the model fit. The following cut-off values were used to establish adequate fit: GFI > 0.90, CFI ≥ 0.95, TLI ≥ 0.95, and RMSEA < 0.06 [30].

## Results

### Sample characteristics

The sample demographic and performance-related characteristics are listed in Table 2.

**Table 2:** Demographic and performance-related characteristics of the students

Variables	Count	%	M ± SD
**Sex**			
Male	47	54.0	
Female	40	46.0	
**Age**			24.76 ± 2.49
23–24 years	57	65.5	
≥ 25 years	30	34.5	
**Academic performance (before clerkship)** ^&^	87		83.16 ± 4.99
**SSC participation**			
Yes	70	80.5	
No	17	19.5	
**SSC outcome**			
Pass	37	52.9	
Fail	33	47.1	
Did not participate	17	n/a	
**OSCE performance**	87		14.67 ± 3.12
**OSCE outcome**			
Pass	67	77.0	
Fail	20	23.0	
**Clerkship performance**	87		85.23 ± 1.20

&: Average scores from first semester of second year to first semester of fifth year

The sample consisted of a higher percentage of males, with ages ranging from 23 to 37 years. OSCE performance scores ranged from 7 to 22 points, while academic performance scores ranged from 70.47 to 91.80 points, and clerkship performance scores ranged from 82.00 to 88.30 points. A majority of students participated in the SSC. Comparisons between students’ participation revealed no significant difference in sex (χ^2^ = 2.334, *p* = 0.127), age (t = 1.372, *p* = 0.0185), and academic performance (t = -1.159, *p* = 0.123).

### SSC outcome analyses

Table 3 compares SSC outcomes in 70 students who participated.

**Table 3:** Comparisons of situational-simulation teaching course outcome

Variables	SSC outcome		*P*-value
	Pass (*n* = 37)	Fail (*n* = 33)	
**Sex**			
Male	17 (48.6%)	18 (51.4%)	0.473^a^
Female	20 (57.1%)	15 (42.9%)	
**Age**	24.68 ± 2.46	24.39 ± 2.09	0.610^b^
**Academic performance** ^&^	83.18 ± 4.88	84.01 ± 5.05	0.490^b^
**OSCE performance**	15.57 ± 2.62	14.94 ± 2.89	0.344^b^
**Clerkship performance**	85.25 ± 1.17	85.49 ± 1.08	0.395^b^

&: Average scores from first semester of second year to first semester of fifth year; a: Chi-square test; b: Independent sample *t*-test; OSCE: objective structured clinical examination; SSC: situational simulation course

Among them, 37 (52.9%) passed the SSC course and 33 (47.1%) failed. The course performance outcomes of 35 (50.0%) males and 35 (35.0%) females were not significantly different based on age, academic performance, OSCE performance, and clerkship performance.

Concerning students’ attitudes toward the SSC course, all five dimensions showed positive attitudes. The average sum score of the situational simulation planning subscale was 24.13 (SD = 1.512) points, ranging from 20 to 25 points; that of the supervision and teaching subscale was 29.21 (SD = 1.693) points, ranging from 24 to 30 points; that of the improvement of self-efficacy subscale was 19.47 (SD = 1.126) points, ranging from 16 to 20 points; that of the interdisciplinary learning subscale was 23.89 (SD = 1.732) points, ranging from 20 to 25 points; and that of the professionalism subscale was 14.47 (SD = 1.126) points, ranging from 9 to 15 points. The five subscales were rated fully by at least half of the students (67.1%, 74.3%, 77.1%, 61.4%, and 75.7%, respectively). Table 4 shows the comparisons of the five subscales, which revealed no statistically significant differences in sex, age, and SSC outcome.

**Table 4:** Comparison of attitudes toward SSC (independent sample *t*-test)

Variables	*n* (%)	M ± SD				
		Situational simulation planning	Supervision and teaching	Improvement of self-efficacy	Interdisciplinary learning	Professionalism
**Sex**						
Male	35 (50.0)	23.86 ± 1.70	29.00 ± 2.00	19.31 ± 1.26	23.66 ± 1.94	14.26 ± 1.40
Female	35 (50.0)	24.40 ± 1.27	29.43 ± 1.31	19.63 ± 0.97	24.11 ± 1.49	14.69 ± 0.72
P-value		0.135	0.293	0.246	0.273	0.113
**Age**						
23–24 years	48 (68.6)	24.06 ± 1.51	29.17 ± 1.64	19.44 ± 1.15	23.85 ± 1.69	14.50 ± 0.97
≥ 25 years	22 (31.4)	24.27 ± 1.55	29.32 ± 1.84	19.55 ± 1.10	23.95 ± 1.86	14.41 ± 1.44
P-value		0.593	0.731	0.712	0.824	0.756
**SSC outcome**						
Pass	37 (52.9)	34.32 ± 1.29	29.43 ± 1.35	19.59 ± 0.96	24.05 ± 1.47	14.62 ± 0.83
Fail	33 (47.1)	23.91 ± 1.72	28.97 ± 2.01	19.33 ± 1.29	23.70 ± 1.99	14.30 ± 1.38
P-value		0.263	0.257	0.336	0.402	0.254

SSC: situational simulation course

The students reported that the SSC effectively improved their communication and comprehension skills (100.0%), ability to apply professional knowledge and skills (92.9%), and empathy and teamwork (81.4%).

### Related factors of OSCE performance

The chi-square test revealed that age, the SSC outcome, and the score of clerkship performance had no significant association with OSCE outcome. However, sex, academic performance, and participation in the SSC showed a significant association (Table 5).

**Table 5:** Factors related to OSCE outcome

Variables	OSCE outcome		*P*
	Pass (*n* = 67)	Fail (*n* = 20)	
**Sex**			
Male	32 (68.1)	15 (31.9)	**0.032** ^**a**^
Female	35 (87.5)	5 (12.5)	
**Age**			
23–24 years	47 (82.5)	10 (17.5)	0.096 ^a^
≥ 25 years	20 (66.7)	10 (33.3)	
**Academic performance** ^&^	84.13 ± 4.66	79.92 ± 4.78	**0.001** ^**b**^
**SSC participation**			
Yes	60 (85.7)	10 (14.3)	**< 0.001** ^a^
No	7 (41.2)	10 (58.8)	
**SSC outcome**			
Pass	33 (89.2)	4 (10.8)	0.379 ^a^
Fail	27 (81.8)	6 (18.2)	
Did not participate	n/a	n/a	
**Clerkship performance**	85.33 ± 1.08	84.89 ± 1.51	0.145 ^b^

&: Average scores from first semester of second year to first semester of fifth year; a: Chi-square test; b: Independent sample *t*-test; OSCE: objective structured clinical examination; SSC: situational simulation course

### Related factors of clerkship performance

The independent sample t-test revealed that sex, age, the SSC outcome, and OSCE performance showed a non-significant association with clerkship performance; however, participation in the SSC and academic performance showed statistically significant differences (Table 6).

**Table 6:** Factors related to clerkship performance

Variables	*n*	Mean ± SD	t	*P*
**Sex**				
Female	40	85.4 ± 1.01	1.270	0.208
Male	47	85.1 ± 1.33		
**Age**				
23–24 years	57	85.3 ± 1.13	1.219	0.226
≥ 25 years	30	85.0 ± 1.31		
**Academic performance** ^&^				
≥ 83 points	47	85.6 ± 1.20	2.929	**0.004**
< 83 points	40	84.8 ± 1.09		
**SSC participation**				
Yes	70	85.4 ± 1.39	-2.235	**0.028**
No	17	84.7 ± 1.30		
**SSC outcome**				
Pass	37	85.3 ± 1.17	0.857	0.395
Fail	33	85.5 ± 1.11		
**OSCE outcome**				
Pass	67	85.3 ± 1.08	-1.471	0.145
Fail	20	84.9 ± 1.51		

&: Average scores from first semester of second year to first semester of fifth year; OSCE: objective structured clinical examination; SSC: situational simulation course

### Path analysis model

**Fig. 2:**
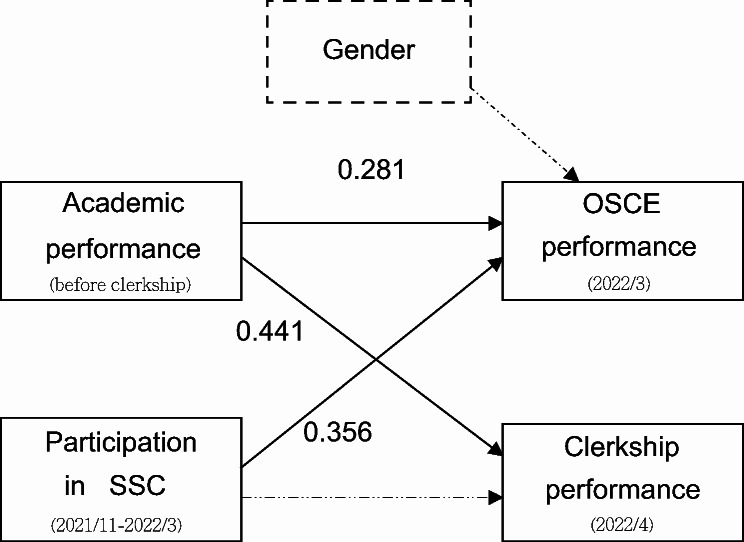
Path analysis model relating academic performance, situational simulation teaching, clerkship performance, and OSCE performance. Standardized path coefficients are presented. Dashed lines represent non-significant paths. OSCE, objective structured clinical examination

A proposed path model was constructed based on the results of a literature review and univariate analyses in this study. Figure 2 illustrates the results of the path-analysis model. The model fit to the data was satisfactory, with the following values: χ^2^ = 2.493, df = 2, *P* = 0.287; RMSEA = 0.054; GFI = 0.986; TLI = 0.965; and CFI = 0.988. The model revealed that academic performance directly affects OSCE performance (β = 0.281, *P* = 0.003) and clerkship performance (β = 0.441, *P* < 0.001); participation in situational simulation teaching course directly affects OSCE performance (β = 0.356, *P* < 0.001).

## Discussion

The SSC was developed based on simulation-based education. In a simulated environment, learners are required to retrieve prior knowledge from their long-term memory and organize this information to engage in problem-solving (cognitivism). Debriefing and reflection sessions enhance this deep learning process, and lead to development of competency. During the class, learners can share, discuss, and learn from others through collaborative learning (constructivism). The SSC emphasizes active learning over passive methods, utilizing simulated environments to replicate actual clinical setting, with students actively participating and receiving feedback from facilitators [17]. In this study, all students (100%) who participated in the course reported an improvement in their ability to communicate and understand patients’ needs. After completing the course, all students exhibited positive attitudes toward the course, with positive feedback across all five dimensions (situational simulation planning, supervision and teaching, self-efficacy improvement, interdisciplinary learning, and professionalism). The course encouraged students to identify their areas for improvement and enhanced their overall learning effectiveness. Previous studies have reported that students preferred a kinesthetic learning style involving physical activity, such as simulation, over traditional lectures [31]. Compared to traditional lectures primarily focusing on knowledge acquisition, the simulation course was effective at enhancing students’ self-efficacy in communication skills and bringing them closer to understanding their professional role [32, 33].

Our results also demonstrated that students who participated in the simulation course performed better on the OSCE, and the path analysis model indicated that the course had a direct impact on OSCE performance. In our previous research, we reported that quite a few of the fifth-year students who attended their first OSCE struggled with interpersonal communication [9]. The present study confirmed that the course improved students’ communication skills, with 85.7% of those who attended the situational simulation teaching course successfully passing the OSCE. This outcome indicates that the SSC could improve students’ non-cognitive skills during the OSCE, addressing the challenges we encountered in the educational setting. The OSCE is a stressful experience for students, and SSC could lead to lower levels of anxiety and improved confidence. Repeated practice and experience have positive effects on OSCE performance [34]. These findings are consistent with those of previous studies that reported positive evaluations of communication skills programs [16, 22, 23, 35]. Our faculty have made efforts to promote the development of dental students’ communication competencies and implemented a situational simulation teaching course to provide students with opportunities to learn and practice these skills. Interestingly, students who gained additional experience post-course demonstrated improved performance in the OSCE, even if their performance during the course was not initially high. This study also found that students’ prior academic performance had no association with their SSC outcomes. However, participation in SSC had an impact on their OSCE performance. This indicated that the opportunities to be exposed to more authentic environments provided by SSC enabled students to engage in experiential learning and develop competency, regardless of variations in their prior knowledge and achievements [17]. Moreover, our study found significant sex differences between OSCE performances in the chi-square test, which is consistent with the results of some previous studies [36], but inconsistent with those of others [9, 37]. However, in the path analysis, these differences were not significant. This evidence suggests that the influence of sex on OSCE performance is not as substantial as that on the SSC. Specifically, SSC can mitigate sex differences in OSCE performance. Previous studies [36] have suggested that educators consider sex-specific teaching strategies to address differences between male and female students. However, this study found that the SSC eliminated sex differences.

The path analysis model revealed that participation in SSC had no impact on clerkship performance, possibly because the clerkship score adopted in this study was the average score assessed by various tutors, each with differing standards across different specialties. Moreover, clerkship training encompasses operational skills, medical knowledge, and non-operational skills, whereas, SSC teaching primarily focuses on non-operational aspects. Conversely, students’ academic performance prior to clerkship directly influenced both their OSCE and clerkship performance. According to Miller’s pyramid of clinical competence, the academic performance of students is represented at the lowest level of the pyramid by “knowledge”. The assessments conducted in SSC and OSCE measure students at the “shows how” level, while clerkship performance represents the highest level of the pyramid by “does.” As students progress from novice (bottom) to expert (top), their learning trajectory should evolve accordingly [38, 39]. The Miller’s Pyramid is suitable to explain our research findings. Although academic performance showed no association with SSC outcomes in our study, it directly impacted students’ performance at the mid (OSCE) and top level (clerkship). Participation in SSC improved the OSCE performance, as both assessments were at the ‘shows how’ level. Previous studies underscore the importance of a robust knowledge foundation during OSCEs: the more knowledge students possess, the better they tend to perform [40, 41].

This study had certain limitations. Firstly, the sample size was restricted to a single university class size; secondly, there may have been a selection bias, as students who volunteered for the study might have had more interest in innovative teaching activities than those who did not. Given that majority of students (80.5%) chose to participate in SSC, there were insufficient students to serve as a control group in this study. Thirdly, due to the inadequate sample size for exploratory factor analysis, we were unable to assess the scale’s construct validity. Nonetheless, student engagement has been shown to promote better learning outcomes. Therefore, further studies involving larger sample sizes or across multiple colleges are needed to investigate these issues among dental students comprehensively.

## Conclusions

SSC can enhance dental students’ non-operational clinical competency and OSCE performance effectively. Simulated patient encounters with feedback in the dental curricula have led to improved communication. Based on our findings, we suggest implementing SSC teaching before the OSCE to improve communication and cognitive skills in dentistry trainees.

## Data Availability

The datasets generated and/or analyzed during the current study are not publicly available because of the regulation of KMUHIRB but are available from the corresponding author upon reasonable request.

## Abbreviations

SP: standardized patient
SSC: situational simulation course
OSCE: objective structured clinical examination
GFI: goodness-of-fit index
CFI: comparative fit index
TLI: Tucker-Lewis index
RMSEA: root mean square error of approximation
